# Targeting Non-Replicating *Mycobacterium tuberculosis* and Latent Infection: Alternatives and Perspectives (Mini-Review)

**DOI:** 10.3390/ijms222413317

**Published:** 2021-12-10

**Authors:** Anna Egorova, Elena G. Salina, Vadim Makarov

**Affiliations:** 1 The Federal Research Centre “Fundamentals of Biotechnology” of the Russian Academy of Sciences (Research Center of Biotechnology RAS), 119071 Moscow, Russia; anna.p.egorova@gmail.com (A.E.); elenasalina@yandex.ru (E.G.S.); 2 Department of Biology and Biotechnology “Lazzaro Spallanzani”, University of Pavia, 27100 Pavia, Italy

**Keywords:** latent tuberculosis infection, dormant *Mycobacterium tuberculosis*, LTBI treatment, anti-TB drugs

## Abstract

Latent tuberculosis infection (LTBI) represents a major challenge to curing TB disease. Current guidelines for LTBI management include only three older drugs and their combinations—isoniazid and rifamycins (rifampicin and rifapentine). These available control strategies have little impact on latent TB elimination, and new specific therapeutics are urgently needed. In the present mini-review, we highlight some of the alternatives that may potentially be included in LTBI treatment recommendations and a list of early-stage prospective small molecules that act on drug targets specific for *Mycobacterium tuberculosis* latency.

## 1. Introduction

Tuberculosis (TB), an infection caused by the bacillus *Mycobacterium tuberculosis*, still remains one of the top 10 causes of death worldwide, especially in low- and lower-middle-income countries [1]. Although TB is preventable and curable, the World Health Organization (WHO) estimates that 7.1 million people fell ill and 1.4 million died from the infection in 2019 [2]. This situation is a result of many factors, such as late diagnosis, co-infection with HIV, and the emergence of multidrug- and extensively drug-resistant (MDR- and XDR-TB) bacilli due to incomplete or inappropriate care. Approximately 0.22 million (3%) of the total new TB cases in 2019 were associated with resistant forms of the infection [2]. However, despite a small share of total TB cases, the number of MDR-TB and XDR-TB cases is increasing every year (0.22 million in 2019 up from 0.20 million in 2018, and 0.17 million in 2017) [2,3,4].

Another global challenge in TB management is the ability of *M. tuberculosis* to cause latent infection, which can suddenly turn into active disease during the lifetime of infected individuals. LTBI may be defined as infection in which tubercle bacilli are persistent in the host but do not currently cause active disease. Latent TB infection is diagnosed by the absence of clinical symptoms and signs and by positive tuberculin skin (TST) or interferon-gamma release assay (IGRA) blood tests. However, these tests/diagnostic approaches have some limitations [5]. Since there is no gold-standard test for LTBI, the exact number of LTBI cases is difficult to establish [6]. It is thought that one fourth of the world’s population is latently infected with *M. tuberculosis*, indicating a large reservoir of individuals with a potential risk of developing active TB [7]. The WHO recommends LTBI treatment for people living with HIV, for people who have household contacts with patients with bacteriologically confirmed pulmonary tuberculosis, and for specific groups of patients receiving immunosuppression therapy (anti-TNF treatment, etc.) [8]. Notably, the likelihood of LTBI activation for these categories may increase from 5% to 10% per year throughout their lifetime. Therefore, addressing LTBI is one of the End TB Strategy crucial milestones for TB elimination [3].

The development of small molecules that suppress latent tuberculosis infection is a non-trivial task, since target-based approaches have had little success. Thus, testing candidate molecules on bacterial cells rather than on isolated enzymes may be the preferred method for finding new drugs for TB as it allows the testing of antibacterial molecules under the appropriate physiological state of the pathogen [9,10]. Therefore, reliable and adequate modeling of latent TB both in vitro and in vivo to search for anti-latent TB drugs is currently a crucial task. Although animal models of LTBI (guinea pigs, mice, rabbits, and non-human primates) may greatly simplify the study of the pathogenic mechanisms of *M. tuberculosis* [11,12,13,14,15,16], due to their relatively high cost and ethical considerations they are unlikely to be applied for primary small molecule screening. Obviously, in vitro models cannot comprehensively reproduce all complex host/pathogen interactions involved in the latent stage of TB, but they are cheaper and save time [17]. Several in vitro models that attempt to mimic latency have been reported [18,19,20,21,22,23,24,25].

## 2. Differences between the Definitions of Latency, Persistence, and Dormancy of *Mycobacterium tuberculosis*

The clinical term latency refers to the asymptomatic state of the infection. LTBI is currently diagnosed by using a tuberculin skin test (TST), which measures delayed-type immune response to a purified protein derivative derived from tuberculin and injected under the skin, or by using an interferon-gamma release assay (IGRA), which measures immune response to the tubercle bacteria in whole blood [26]. The term latency was introduced by Clemens von Pirquet, who in 1907 developed the tuberculin test applied to the skin by scarification [27]. It is believed that culture of tubercle cells from sputum or other samples of a latently infected person is impossible, indicating a low bacterial load in such a patient [26].

The term persistence is mostly used to describe the ability of *M. tuberculosis* to survive and persist in host tissues under stress. Concerning bacterial infections, the term was first introduced in 1944 by Joseph Bigger to characterize a small subgroup in a growing population of staphylococci that was genetically susceptible to drugs but was able to persist after long-term treatment with penicillin [28]. McDermott defined *Mycobacterium tuberculosis* persistence as the “capacity of drug-susceptible microorganisms to survive drug attacks when subsisting in an animal body” [29]. Thus, persistence arises under the effect of antibiotics, while latency is the result of the host’s immune system activity.

The term dormancy comes from the Latin word “dormire” (“to sleep”) and describes a bacterial phenotype with reduced metabolism and slower cell division [17]. This term is often used in the context of the in vitro model of progressive hypoxia (the Wayne model), where *M. tuberculosis* cells decrease metabolism and replication rate, and become phenotypically resistant to isoniazid [18].

## 3. Pathogenesis of Latent Tuberculosis Infection in Humans

Tubercle bacilli that cause LTBI were traditionally assumed to represent a population of dormant non-replicating bacilli with a temporary inability to grow and divide, and with reduced metabolic activity as a result of an adaptive response to immune-mediated mechanisms [30]. Lillebaek et al. showed that the restriction fragment length polymorphism (RFLP) associated with the insertion sequence profiles of latent isolates has not changed for decades, supporting the idea of reduced bacterial replication in the latent state [31]. Colangeli et al. also demonstrated no increase in the number of mutations using whole genome sequencing of *M. tuberculosis* isolates when comparing cases from over 20 years ago and the present day [32]. So, both latent and active tuberculosis are considered to be caused by a heterogeneous population of mycobacteria, including both actively growing bacilli and bacilli with reduced metabolic activity, only in different proportions [33].

Generally, the pathogenesis of LTBI in humans is characterized by a range of features [34]. Primary granulomas, a pathological hallmark of tuberculosis, are formed mostly in the basis pulmonis under the influence of very small amounts of tubercle bacilli (from 1 to 5). In primarily infected persons, the lesions most often disappear spontaneously and asymptomatically, but a local or systemic disease develops in 5–10% of infected persons (mostly in children) in the next 1–2 years [35]. In more than 90% of cases, tuberculosis infection is latent and asymptomatic; after 3–8 weeks of primary infection, the tuberculin skin test becomes positive—this status persists throughout life. *Mycobacterium tuberculosis* cells may also migrate with lymph and blood from primary lesions to secondary locations in the apical regions of the lungs where post-primary granulomas form (post-primary TB) [36]. For reasons that remain unclear, in about 10% of cases of the post-primary disease, the host’s immune system is unable to control the infection. Dormant bacteria begin to reactivate and actively divide, which leads to an increase in their concentration in granulomas in the apical regions of the lungs. Taken together, the infection–disease–infection cycle, mediated by the reactivation of dormant *M. tuberculosis* cells in TST-positive people with latent TB disease, is precisely the mechanism by which *M. tuberculosis* maintains its survival and spreads to new hosts.

## 4. In Vitro and In Vivo Models to Imitate *Mycobacterium tuberculosis* Latency

The Wayne model, developed in 1996, is probably the most studied model of *M. tuberculosis* dormancy [18]. The idea is based on the gradual oxygen depletion of tubercle bacilli, in which dormant cells are obtained by adaptation to hypoxia. Limitations of the model include the lack of explanation for other aspects of dormancy, such as bacterial adaptation to other stresses; the possibility of growing immediately after recovery; and lack of standardization of the method [37]. Another model that utilizes hypoxia is the low-oxygen recovery assay (LORA) [38]. The model uses a recombinant *M. tuberculosis* luciferase reporter to provide results of compound activity against non-replicating tubercle bacilli surviving under hypoxic conditions.

Almost 90 years ago, Loebel et al. noticed that nutritional deficiencies lead to decreased growth and metabolic activity of mycobacteria [39]. Much later, Betts et al. modified the Loebel nutrient-deficient model to design a simple method for testing molecules active against persistent bacteria [20]. The principle is based on the complete deprivation of nutrients from a culture medium. To achieve this, the nutrient-rich culture medium is washed with phosphate-buffered saline, which leads to a gradual shutdown of respiration and a switch of viable bacilli to a dormant state. Like the Wayne model, the Loebel–Betts model also cannot comprehensively mimic the environment of granuloma.

The multiple-stress model was designed to overcome the limitations associated with single-stress conditions. The model proposed by Deb et al. uses a low-nutrient medium that has an acidic pH of 5.0 with a gas mixture of low oxygen and high carbon dioxide (5% O_2_ + 10% CO_2_ + 85% N_2_) [22]. The streptomycin-dependent model represents another in vitro model of *M. tuberculosis* dormancy [40]. The *M. tuberculosis* strain underlying the model was isolated from the sputum of a TB patient who was resistant to streptomycin therapy in Japan in 1955 [41]. The *M. tuberculosis* strain ss18b is characterized by an inability to grow in vitro in the absence of streptomycin [40]. Although the bacteria could not divide, they maintained their viability and ability to reproduce after adding streptomycin to the culture medium.

There are several in vivo models of latent infection that are currently used to explain the disease development. Among them, zebrafish is the cheapest and simplest model that mimics *Mycobacterium tuberculosis* latency, and it may be used in the early preclinical stages of drug discovery [42]. However, most models of TB latency have been developed in mice: the chronic tuberculosis model [43]; the Cornell model based on the treatment of *M. tuberculosis*-infected mice with antibiotics (isoniazid and pyrazinamide), as a result of which bacilli are not detected by organ culture [11]; the artificial granuloma model [44]; and the Kramnik model, in which specially grown C3HeB/FeJ mice demonstrated the key features of latent tuberculosis in humans [13]. In guinea pig/rabbit models, granulomas are very similar to those in humans, but these animals are highly susceptible to rapid disease progression [45]. The most useful animal model of LTBI is the non-human primate model—this model most accurately reproduces the clinical, histological, and microbiological characteristics of latent infection in humans [46]. Limitations of the model include high cost and ethical considerations. Although there is not an ideal model, these animal models are used to study the efficacy of molecules active against dormant mycobacteria.

## 5. Alternatives for Latent Tuberculosis Infection Treatment

Since specific and effective anti-latent TB drugs are still in short supply, traditional antituberculosis drugs—isoniazid, rifampicin, and rifapentine—are used in modern LTBI chemotherapy, and therapeutic regimens are generally based on long-term treatment of latently infected patients with these drugs [6,47,48]. Isoniazid obviously has low efficiency against latent TB infection because it targets bacterial cell wall biosynthesis, which is inactive during dormancy [49]. The role of isoniazid in LTBI therapy is probably to kill emerging bacilli that grow actively as a result of reactivation of dormant cells or transformation of slowly growing persisters into actively growing bacilli susceptible to INH treatment [49]. Rifampicin is used as an alternative agent to reduce the side effects of isoniazid therapy (e.g., hepatotoxicity) [47]. Rifapentine, in turn, has a longer half-life and greater potency against *M. tuberculosis* than rifampicin [50]. Rifamycins block transcription by binding to the b-subunit of RNA polymerase—an important stage in the life of actively growing mycobacteria—so the effectiveness of the above drugs in the treatment of LTBI is still an open question.

Three anti-TB drugs with original targets have the potential to be selected in LTBI treatment guidelines. Bedaquiline (formerly TMC207 or R207910) was approved to treat active tuberculosis in 2012, being the first anti-TB drug discovered in the past 40 years. The molecule targets mycobacterial F_0_F_1_ ATP synthase and thus stops the production of ATP required for cellular energy production [51]. In further study, Andries’ research group observed that TMC207/R207910 treatment leads to a 1.8-log reduction in CFU in the Wayne model and 2.1-log in CFU in the hypoxia-induced model [52]. A killing kinetic study revealed that the compound completely sterilizes dormant bacilli in the Wayne hypoxia model in vitro after 14 days. Andries et al. proposed that ATP synthase contributes to mycobacterial survival despite its downregulation during the latent state and thus may potentially serve as a target for dormant bacteria. Rao et al. also confirmed TMC207/R207910 is highly active against non-replicating mycobacteria under hypoxic conditions [53]. The in vivo efficacy of bedaquiline was determined in several murine models of LTBI [54,55].

Pretomanid (formerly PA-824), belonging to the chemical class of nitroimidazoles, was initially identified as an attractive small molecule for TB treatment in 2000 and was approved for clinical use in 2019 by the U.S. Food and Drug Administration (FDA) and in 2020 by the European Medicines Agency (EMA) [56]. The compound exhibits bactericidal activity not only against actively replicating *M. tuberculosis*, but also against non-replicating bacteria in the Wayne hypoxia model [56,57]. PA-824 is a prodrug, which is converted to an active metabolite by deazaflavin-dependent nitroreductase (Ddn). Singh et al. suggested that the des-nitro metabolite and reactive nitrogen species, primarily nitric oxide (NO) generated from it, significantly contribute to PA-824’s anaerobic activity against non-replicating tubercle bacilli [58]. To verify the in vivo efficacy of pretomanid against dormant *M. tuberculosis*, Dutta and Karakousis used a necrotic granuloma murine model of LTBI, which is considered a highly clinically relevant model [59,60]. PA-824 demonstrates relatively limited efficacy against latent *M. tuberculosis* infection in C3HeB/FeJ mice, reducing bacterial load from Month 1 to Month 4 by only ~1.0-log CFU [60]. Another anti-TB nitroimidazooxazole derivative, FDA-approved delamanid (formerly OPC-67683), has also been investigated against LTBI. Chen et al. reported that the molecule kills more than 50% of the population of dormant bacilli in a modified Wayne model at concentrations of 0.4 mg/L and above [61]. In *M. tuberculosis*-infected guinea pigs, delamanid completely eradicates bacterial CFU in lungs over time (2.04-log CFU at 4 weeks, 0-log CFU at 8 weeks) compared with the control (5.91-log CFU at 4 weeks, 5.64-log CFU at 8 weeks) [61].

A few drugs from other therapeutic areas are being investigated as repurposable alternatives for LTBI treatment. For example, tafenoquine, an antimalarial agent from the 8-aminoquinoline class, was found to be active in a nutrient-starved dormancy model with a ~2.0-log decrease in CFU [62]. Other works evaluate derivatives of sacubitrile, an antihypertensive drug used in combination with valsartan, and pleuromutilin, an antibiotic, against dormant tubercle cells [63,64].

## 6. Early-Stage Compounds Active against Dormant and Non-Replicating Bacilli

The search for small-molecule inhibitors of dormant *M. tuberculosis* is a challenging process. First of all, dormant bacilli are characterized by reduced metabolism, and therefore most bacterial targets are non-druggable and only a few *M. tuberculosis* enzymes may be inhibited. In addition, all currently available in vivo and in vitro models of TB dormancy have certain limitations, such as high metabolic activity of dormant mycobacteria, lack of difference between the clinical manifestations of active and dormant TB, or an ambiguous assessment of the reduction in bacterial load. However, despite the existing obstacles, a number of recently discovered small molecules with anti-latent TB activity may be exploited as a platform for further in-depth study of the prospective *M. tuberculosis* molecular targets for LTBI elimination.

### 6.1. Dormancy Survival Two-Component Regulatory System (DosRST)

**Figure 1:**
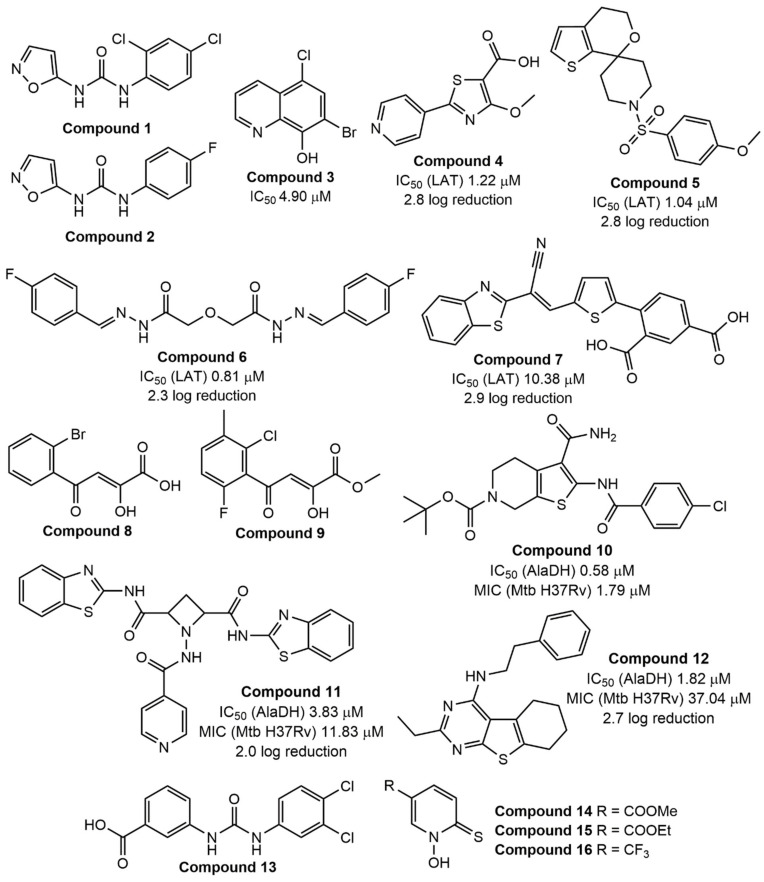
Early-stage small molecules with established drug targets for dormant *M. tuberculosis*. Compound 1: 1-(2,4-dichlorophenyl)-3-(isoxazol-5-yl)urea; compound 2: 1-(4-fluorophenyl)-3-(isoxazol-5-yl)urea; compound 3: 7-bromo-5-chloroquinolin-8-ol; compound 4: 4-methoxy-2-(pyridin-4-yl)thiazole-5-carboxylic acid; compound 5: 1-((4-methoxyphenyl)sulfonyl)-4′,5′-dihydrospiro[piperidine-4,7′-thieno[2 ,3-*c*]pyran]; compound 6: 2,2′-oxybis(*N*′-(4-fluorobenzylidene)acetohydrazide); compound 7: 4-(5-(2-(benzo[*d*]thiazol-2-yl)-2-cyanovinyl)thiophen-2-yl)isophthalic acid; compound 8: 4-(2-bromophenyl)-2-hydroxy-4-oxobut-2-enoic acid; compound 9: methyl ester of -4-(2-chloro-6-fluoro-3-methylphenyl)-2-hydroxy-4-oxobut-2-enoic acid; compound 10: *tert*-butyl ester of 3-carbamoyl-2-(4-chlorobenzamido)-4,7-dihydrothieno[2,3-*c*]pyridine-6(5*H*)-carboxylic acid; compound 11: *N*^2^,*N*^4^-bis(benzo[*d*]thiazol-2-yl)-1-(isonicotinamido)azetidine-2,4-dicarboxamide; compound 12: 2-ethyl-*N*-phenethyl-5,6,7,8-tetrahydrobenzo[4,5]thieno[2,3-*d*]pyrimidin-4-amine; compound 13: 3-(3-(3,4-dichlorophenyl)ureido)benzoic acid; compound 14: methyl ester of 1-hydroxy-6-thioxo-1,6-dihydropyridine-3-carboxylic acid; compound 15: ethyl ester of 1-hydroxy-6-thioxo-1,6-dihydropyridine-3-carboxylic acid; compound 16: 1-hydroxy-5-(trifluoromethyl)pyridine-2(1*H*)-thione.

Low oxygen level (hypoxia), nutrient starvation, acidic pH, and other environmental and host immune pressures force active mycobacterial cells to switch into a dormant state with reduced metabolism [65]. *M. tuberculosis* is known to exploit DosRST to regulate its dormancy and virulence, and, especially, to promote survival during this state [66,67,68,69]. The DosRST regulatory system consists of two heme-based histidine sensor kinases, DosS and DosT, and the response regulator DosR, which are activated under stress conditions [70]. Given the importance of this system for maintaining dormancy in mycobacteria, it may be an attractive target for the control of LTBI. The feasibility of this approach for suppressing tubercle bacilli was first explored by Gupta et al. in 2009 [71]. Later, Zheng et al. screened a large chemical library using the DosRST regulon fluorescent *M. tuberculosis* reporter strain CDC1551 [72]. They suggested that inhibition of such fluorescence may be associated with inactivation of the DosRST. As a result, HC106A (compound 1, Figure 1) was found to inhibit dosR-dependent GFP fluorescence with an EC_50_ of 6.9 μM or 2.5 μM, and further to modulate the DosRST signaling by directly targeting the heme sensor [72]. To understand the structure–activity relationships (SAR) of the hit compound, a range of urea derivatives with different substitution patterns were further synthesized [72]. The isoxazole ring was important for the function as its replacement with another hetero/aryl group led to a decrease in fluorescence inhibition. The urea moiety was also needed for better activity. Particular attention has been paid to the role of the 2,4-dichlorophenyl ring. So, 1-(4-fluorophenyl)-3-(isoxazol-5-yl)urea (compound 2, Figure 1) was selected as the most active inhibitor of DosRST with an EC_50_ of 0.54 μM.

### 6.2. Methionine Aminopeptidase (MetAP)

Bacterial methionine aminopeptidases are metalloenzymes that catalyze the cleavage of amino acid residues at the *N*-terminal position of peptides and proteins [73]. To validate mycobacterial metalloproteinase (MtMetAP) as a druggable target, Olaleye et al. screened a chemical library and found that inhibitors of these mycobacterial enzymes were active against both replicating and aged non-growing (non-replicating) *M. tuberculosis* in the Byrne model of persistence [74,75]. Continuing this research, they characterized 7-bromo-5-chloroquinolin-8-ol CLBQ14 (compound 3, Figure 1), a bromine analog of clioquinol, as a suitable inhibitor of non-replicating bacilli [76]. Clioquinol is an antiprotozoal drug from the 8-hydroxyquinoline class that is probably neurotoxic at high doses. Olaleye et al. hypothesized that CLBQ14 does not show the same adverse effect, but they did not confirm or reject this point of view. Moreover, in recent preclinical studies of this compound in rats, the researchers also did not provide a toxicity profile of CLBQ14 [77].

### 6.3. Lysine ε-Aminotransferase (LAT)

Lysine ε-aminotransferase (LAT), a pyridoxal 5′-phosphate (PLP)-dependent enzyme, was found to be involved in the formation of persisters in mycobacteria [78,79]. A little earlier, Betts et al. determined that this enzyme is activated ~42-fold in their own developed nutrient-starved model of dormancy [20]. To understand the potential of LAT as a therapeutic target, Sriram et al. selected a number of primary hits using an e-pharmacophore approach and synthesized a library of hit-based analogs to assess in vitro activity [80,81,82,83]. Some interesting second-generation hits are presented in Figure 1. Three of the four compounds exhibit acceptable activity towards the target in an LAT enzyme inhibition assay (except for compound 7) together with good bacterial log reduction in a nutrient-starved model of *M. tuberculosis* dormancy.

### 6.4. Isocitrate Lyase (ICL)

Isocitrate lyases (ICLs) represent Mg^2+^-dependent enzymes that catalyze the reversible cleavage of D-isocitrate to glyoxylate and succinate in the glyoxylate shunt [84]. Actually, *M. tuberculosis* contains two isocitrate lyases with 27% sequence identity, ICL1 (428aa) and ICL2 (766aa), encoded by the *icl1* and *aceA* genes, respectively [85]. Evidence indicates that ICLs are essential proteins not only for virulence [86,87], but also for the survival of non-replicating *M. tuberculosis* in a nutrient-deprived, oxygen-rich model of latency (the Loebel–Betts model) [88]. Several compounds have been investigated to validate the druggability of the target. 3-Nitropropionate and itaconate (structures not shown) strongly inhibit *M. tuberculosis* ICLs and, however, were found to be very toxic [89,90]. Bhusal et al. defined the main problems in the development of ICL inhibitors: the highly polar nature of the ICL binding pocket may lead to a drug selectivity issue, and the small size of the pocket will not allow a complete SAR study of the small molecules [91]. Some other studies have identified 4-(4-methoxy-phenyl)-4-oxo-crotonic acid methyl ester (IMBI-3), phenolic *N*-mono-substituted carbamates, salicylanilide derivatives, and cis-2,3-epoxy-succinic acid (structures not shown) as ICL inhibitors; however, limited data do not permit a conclusion about the usefulness of their further development [92,93,94,95].

### 6.5. Malate Synthase (GlcB)

While isocitrate lyase catalyzes the first step of the glyoxylate shunt, malate synthase acts in the second step of this pathway and converts glyoxylate into malate using one molecule of acetyl-CoA [96,97]. Krieger et al. suggested that GlcB may be a more “druggable” target than ICL [96]. Using a focused library of glyoxylate-like small molecules, researchers identified (Z)-2-hydroxy-4-oxo-4-phenylbut-2-enoic acid (PDKA) as a suitable GlcB inhibitor with an IC_50_ value of 2.0 µM [96]. However, PDKA was unstable in cell growth media (half-life t_1/2_ = 3 days). The PDKA derivative containing an ortho-bromine atom in the 4-phenyl ring (compound 8, Figure 1) exhibited a more pronounced enzyme inhibition effect (IC_50_ = 0.6 µM), but it is also not sufficiently stable. At the same time, the derivative with three substituents in the phenyl ring was weaker inhibitor of GlcB (IC_50_ = 5.5 µM) but with a longer half-life. To improve stability, Krieger et al. synthesized and evaluated the pharmacological properties of several alkyl- and benzyl-ester prodrugs of selected PDKA analogs [96]. The methyl ester (compound 9, Figure 1) showed a more favorable in vitro pharmacokinetic profile than other prodrugs and was selected for further detailed studies.

### 6.6. L-Alanine Dehydrogenase (L-AlaDH)

L-Alanine dehydrogenase is another enzyme expressed by mycobacteria during the latent state [98]. This enzyme catalyzes a reversible conversion of L-alanine to pyruvate. Griffin et al. also proposed that L-AlaDH maintains an optimal NADH/NAD ratio during mycobacterial cells’ recovery from hypoxic state (anaerobiosis), and the enzyme may be utilized as a potential target for TB dormancy inhibitors [99]. So, Shiram’s research group attempted to develop L-AlaDH inhibitors [100,101,102]. In the articles discussed, they identify a prospective parent structure from each virtual screening and synthesize a range of molecules based on this backbone. Then, they evaluate the activity of the compounds against nutrient-starved dormant bacilli to investigate the SAR. High enzyme-specificity compounds were cytotoxic and, in contrast, low enzyme-specificity molecules showed less cytotoxic effect. In the nutrient-starved model of dormant *M. tuberculosis*, the bacterial reduction value was approximately 2-log. A few interesting hits (compounds 10–12) are presented in Figure 1. However, the rationale for further optimization of these chemical classes needs to be clarified in detail.

### 6.7. Cysteine Synthase (CysM)

Cysteine synthase (CysM), one of three pyridoxal phosphate-dependent cysteine synthases in *M. tuberculosis*, is responsible for the synthesis of cysteine using O-phosphoserine and a sulfur carrier protein CysO [103]. Moreover, cysteine biosynthesis plays an important role during *M. tuberculosis* dormancy, since cysteine is involved in the biosynthesis of mycothiol, which is used to maintain redox homeostasis by mycobacteria [104]. A diverse library screening campaign by Brunner et al. led to the selection of urea-based hits for follow-up experiments. 3-(3-(3,4-Dichlorophenyl)ureido)benzoic acid (compound 13, Figure 1) shows suitable CysM-specific affinity with a K_d_ value of 0.32 μM and a 3-log decrease in bacterial count in a nutrient-starved model of dormancy (or the Loebel–Betts model) [105]. A preliminary SAR study revealed that the introduction of an additional substituent in the left phenyl ring or the replacement of two chlorine atoms with various groups in the right phenyl ring had little effect on target-specific affinity. At the same time, the urea moiety is important for binding to the CysM active site. Furthermore, Brunner et al. additionally evaluated these urea-based derivatives for binding with two another cysteine synthases, CysK1 and CysK2, and found some interesting inhibitors [106]. However, in-depth SAR investigation is needed to understand the potential of this chemical class as CysM inhibitors.

### 6.8. Copper-Mediated Innate Immunity

Copper-mediated innate immunity and its antibacterial properties [107,108,109,110] coupled with the ability of the metal ion to accumulate in bacteria-infected macrophage phagosomes [111] suggest copper as a viable antibacterial weapon. 1-Hydroxy-5-R-pyridine-2(1H)-thiones (compounds 14–16, Figure 1), active against streptomycin-deficient cells of *M. tuberculosis* 18b strain in vitro [25], form stable, charged lipophilic complexes with Cu^2+^ ions for transport into *M. tuberculosis* cells [112]. Subsequent metabolic transformation of the compounds led to the release and accumulation of free ions in the cytoplasm of bacilli. The actual molecular target of copper toxicity is still debated, but it may be scattered across a range of metabolic targets containing or constructing iron–sulfur clusters [113,114,115]. Hence, copper may actually affect a wide variety of cellular processes; this “target plurality” appears to be effective in inhibiting tubercle bacilli at different metabolic stages, including the dormant state.

### 6.9. Unknown Targets

**Figure 2:**
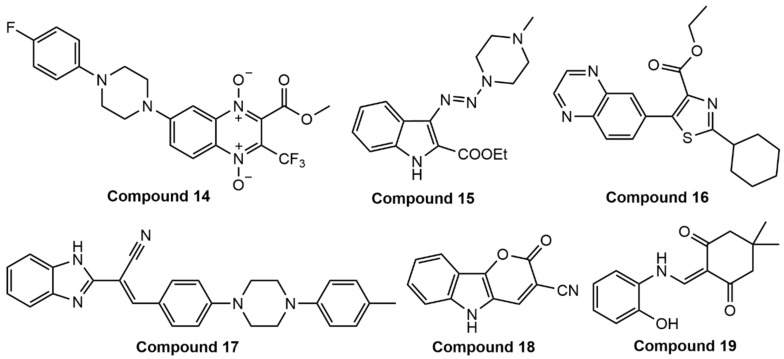
Small molecules active against dormant *M. tuberculosis* by an unknown mechanism of action. Compound 14: 6-(4-(4-fluorophenyl)piperazin-1-yl)-3-(methoxycarbonyl)-2-(trifluoromethyl)quinoxaline 1,4-dioxide; compound 15: ethyl ester of 3-((4-methylpiperazin-1-yl)diazenyl)-1*H*-indole-2-carboxylic acid; compound 16: ethyl ester of 2-cyclohexyl-5-(quinoxalin-6-yl)thiazole-4-carboxylic acid; compound 17: 2-(1*H*-benzo[d]imidazol-2-yl)-3-(4-(4-(p-tolyl)piperazin-1-yl)phenyl)acrylonitrile; compound 18: 2-oxo-2,5-dihydropyrano[3,2-*b*]indole-3-carbonitrile; compound 19: 2-(((2-hydroxyphenyl)amino)methylene)-5,5-dimethylcyclohexane-1,3-dione.

Santivañez-Veliz et al. reported that the quinoxaline-based molecule (compound 14, Figure 2) inhibits *M. tuberculosis* cells with an MIC value of 0.375 µg/mL in a low-oxygen recovery assay (LORA) [116]. Nikonenko et al. synthesized the 3-triazenoindole-based molecule TU112 (compound 15, Figure 2), which inhibits dormant *M. tuberculosis* by ~2.0-log in the Salina model, but the toxicity profile of the molecule and the class as a whole strongly needs to be optimized [117]. In another article, a thiazole derivative (compound 16, Figure 2) was found to show more than 90% inhibition of dormant *M. tuberculosis* H37Ra at 10 µM [118]. Bonnet et al. demonstrated the bactericidal activity of hydrazones (structures not shown) against non-replicating *M. tuberculosis* in two (hypoxia and starvation) dormancy models [119]. A benzimidazole–acrylonitrile hybrid (compound 17, Figure 2) shows a 2.8-log reduction in mycobacterial count in a nutrient-starved dormancy model [120]. Monakhova et al. investigated a class of pyrano[3,2-b]indolones and found that a particular compound (compound 18, Figure 2) exhibits good activity against *M. tuberculosis* H37Rv and the streptomycin-starved *M. tuberculosis* 18b model (ss18b) with MIC_99_ values of 0.3 and 0.4 μg/mL, respectively [121]. Rather et al. evaluated the potency of a recently discovered compound, PAMCHD (compound 19, Figure 2), against *M. tuberculosis* in several in vitro models mimicking the latent state [122]. PAMCHD was found to sterilize persister *M. tuberculosis* cells in a hypoxia-induced model with a 6.5-log CFU decrease, as well as in a nutrient-starved model with a 7.5-log decrease. In some other works, molecular docking has been used to predict drug targets for the compounds discovered [123,124,125,126]. We have included these articles in the present chapter of the review because in all these cases, mechanism-of-action studies are needed to clarify the site of action of the molecules.

## 7. Concluding Remarks and Future Outlook

Latent tuberculosis infection is a serious obstacle to the complete elimination of tuberculosis. Therapeutic and preventive options for LTBI treatment are limited. For example, the only vaccine licensed against TB thus far is Bacille Calmette-Guérin (BCG), which was approved a century ago [127]. BCG vaccination primarily provides consistent protection against the most severe forms of childhood tuberculosis and practically does not provide protection against adult-type tuberculosis [128]. In 2019, GSK reported that the M72/AS01_E_ vaccine candidate reduces the incidence of pulmonary tuberculosis in HIV-negative adults with latent infection. It demonstrated an overall efficacy of 50% for at least three years after vaccination in a Phase IIb study, but there is still no information on additional clinical trials or market launch of the vaccine [129].

Phage therapy may play an important role in TB management. Recently, Guerrero-Bustamante et al. found that a five-phage cocktail minimized the emergence of phage resistance and cross-resistance to multiple phages, and efficiently killed *M. tuberculosis* strains tested [130]. We hypothesize that the evaluation of bacteriophages against dormant tubercle cells represents an interesting research direction, but more data are needed to clarify this point.

The discovery of small molecules acting on both active and dormant *M. tuberculosis* cells seems to be the most promising direction. In the present mini-review, we highlighted early-stage compounds targeting the dormancy survival two-component regulatory system and a number of enzymes, such as methionine aminopeptidase, lysine-aminotransferase, or malate synthase, that are specific for the dormant state of tubercle bacilli. In addition, the discovery of multiple-target compounds that can induce apoptosis only in mycobacterial cells, for example, through copper-mediated innate immunity, may also represent an attractive way to target dormant bacteria.

Finally, tackling the spread of tuberculosis from two ends—small-molecule drug discovery and vaccination—holds great promise for fighting this dangerous and persistent pathogen.
